# Prediction of premature all-cause mortality: A prospective general population cohort study comparing machine-learning and standard epidemiological approaches

**DOI:** 10.1371/journal.pone.0214365

**Published:** 2019-03-27

**Authors:** Stephen F. Weng, Luis Vaz, Nadeem Qureshi, Joe Kai

**Affiliations:** 1 NIHR School for Primary Care Research, University of Nottingham, Nottingham, United Kingdom; 2 Primary Care Stratified Medicine (PRISM), Division of Primary Care, School of Medicine, University of Nottingham, Nottingham United Kingdom; Tabriz University of Medical Sciences, ISLAMIC REPUBLIC OF IRAN

## Abstract

**Background:**

Prognostic modelling using standard methods is well-established, particularly for predicting risk of single diseases. Machine-learning may offer potential to explore outcomes of even greater complexity, such as premature death. This study aimed to develop novel prediction algorithms using machine-learning, in addition to standard survival modelling, to predict premature all-cause mortality.

**Methods:**

A prospective population cohort of 502,628 participants aged 40–69 years were recruited to the UK Biobank from 2006–2010 and followed-up until 2016. Participants were assessed on a range of demographic, biometric, clinical and lifestyle factors. Mortality data by ICD-10 were obtained from linkage to Office of National Statistics. Models were developed using deep learning, random forest and Cox regression. Calibration was assessed by comparing observed to predicted risks; and discrimination by area under the ‘receiver operating curve’ (AUC).

**Findings:**

14,418 deaths (2.9%) occurred over a total follow-up time of 3,508,454 person-years. A simple age and gender Cox model was the least predictive (AUC 0.689, 95% CI 0.681–0.699). A multivariate Cox regression model significantly improved discrimination by 6.2% (AUC 0.751, 95% CI 0.748–0.767). The application of machine-learning algorithms further improved discrimination by 3.2% using random forest (AUC 0.783, 95% CI 0.776–0.791) and 3.9% using deep learning (AUC 0.790, 95% CI 0.783–0.797). These ML algorithms improved discrimination by 9.4% and 10.1% respectively from a simple age and gender Cox regression model. Random forest and deep learning achieved similar levels of discrimination with no significant difference. Machine-learning algorithms were well-calibrated, while Cox regression models consistently over-predicted risk.

**Conclusions:**

Machine-learning significantly improved accuracy of prediction of premature all-cause mortality in this middle-aged population, compared to standard methods. This study illustrates the value of machine-learning for risk prediction within a traditional epidemiological study design, and how this approach might be reported to assist scientific verification.

## Introduction

In the era of big data, there is great optimism that machine-learning (ML) can potentially revolutionise care, offer approaches for diagnostic assessment and personalise therapeutic decisions on a par with, or superior, to clinicians. ML techniques rely on machine-guided computational methods rather than human-guided data analysis to fit a “function” to the data in more standard statistical methods [1]. While ML can still use familiar models such as logistic regression, many other ML techniques do not use a pre-determined equation. Artificial neural networks [2], for example, seeks to determine the “best function” which efficiently models all complex and non-linear interactions between variables while minimising the error between predicted and observed outcomes.

The most prominent area of research which has seen rapid growth in the use of ML is in the field of diagnostics and prognosis [3–7]. Traditionally, prognostic modelling has relied on standard statistical methods, yielding clinical risk prediction algorithms, for example, predicting future risk of cardiovascular disease (CVD) [8, 9]. Many such algorithms demonstrate high predictive accuracy, verified and replicated with numerous validation studies [10]. Thus, the challenge for applications and algorithms developed using ML is to not only enhance what can be achieved with traditional methods, but to also develop and report them in a similarly transparent and replicable way.

Prognostic modelling using standard methods [11] is well-established, particularly for predicting risk of single disease [10]. Our recent research has used ML approaches for prognostic modelling using routine primary care data. This demonstrated improved accuracy for prediction of CVD of around 3.6% compared to standard approaches (by increasing the area under the receiver operating curve [AUC]), and also suggested potential novel risk factors [5]. Machine learning may offer potential to also explore outcomes of even greater complexity and multifactorial causation, such as premature death.

Thus far, no prognostic studies using ML methods with observational data have detailed how ML can be integrated with traditional epidemiological study design. We aimed to develop and report novel prognostic models, using machine-learning methods, in addition to standard survival modelling, to predict premature, all-cause mortality in a large and contemporary population cohort (UK Biobank).

## Methods

### Study population

The UK Biobank is a large prospective population cohort of 502,628 participants aged 40–69 years, recruited between 2006 and 2010 from across the UK, who have consented to have their health followed [12, 13]. Participants were assessed at recruitment centres across the UK via a touchscreen questionnaire and nurse-led interview on a range of socio-demographic, behavioural, nutritional, lifestyle, medication history, and clinical history. Participants had undergone physical assessment, and data collection on biometrics, blood and saliva sample collection for future analysis. Health outcomes were sourced from linkages to the UK cancer registry, Office of National Statistics (ONS) death records, and hospital episodes statistics (HES). The latest linkage used in this study was completed on February 2016; the last wave of participants had a minimum of six years follow-up, while the first wave had up to a maximum of 10 years follow-up. The cohort offers a unique opportunity to study premature mortality as all registered deaths during follow-up, since 2006 when recruitment began, occurred at lower than expected UK population life expectancy.

Approvals to access the anonymised prospective cohort for this study were granted by the UK Biobank Access Management Team (No. 24321). Ethical approval was granted by the national research ethics committee (REC 16/NW/0274) for the overall UK Biobank cohort.

### Baseline variables

Baseline variables were pragmatically identified on biological plausibility, as well as being mostly complete in the baseline questionnaire, with limited missing or unknown values. In total, 60 predictor variables at baseline were included in developing the prediction models for all-cause mortality including demographics, family history, clinical history, medications, lifestyle, diet, supplements, environmental exposure, and biometrics. These variables and coding structure are documented in **Box 1.**

**Box 1. Baseline predictor variables to be considered for inclusion in predicting all-cause mortality**

➢ Age (years)
➢ Gender (female; male)
➢ Educational qualifications (none; College/University; A/AS levels; O levels/GCSEs; CSEs; NVQ/HND/HNC; other professional qualifications; unknown)
➢ Townsend deprivation index (continuous)
➢ Ethnicity (White; South Asian; East Asian; Black; other/mixed race; unknown)
➢ Height (m)
➢ Weight (kg)
➢ Waist circumference (cm)
➢ Body mass index (kg/m^2^)
➢ Body fat percentage (%)
➢ Forced expiratory volume 1 (L)
➢ Diastolic blood pressure (mm HG)
➢ Systolic blood pressure (mm HG)
➢ Skin tone (very fair; fair; light olive; dark olive; brown; black; unknown)
➢ Vitamins and supplements (none; vitamin A; vitamin B; vitamin C; vitamin D; vitamin B9; calcium; multi-vitamins)
➢ Family history of prostate cancer (no; yes)
➢ Family history of breast cancer (no; yes)
➢ Family history of colorectal cancer(no; yes)
➢ Family history of lung cancer (no; yes)
➢ Smoking status (non-smoker; current smoker)
➢ Environmental tobacco smoke (hours per week)
➢ Residential air pollution PM2.5 (quintiles of μg/m^3^)
➢ Physical activity (MET-min per week)
➢ Beta-carotene supplements (no; yes)
➢ Alcohol consumption (never, special occasions only; 1–3 times per month; 1–3 times per week; daily or almost daily, unknown)
➢ Fruit consumption (pieces per day)
➢ Vegetable consumption (pieces per day)
➢ Beef consumption (never; < one per week; one per week; 2–4 times per week; 5–6 times per week; once or more daily; unknown)
➢ Pork consumption (never; < one per week; one per week; 2–4 times per week; 5–6 times per week; once or more daily; unknown)
➢ Processed meat consumption (never; < one per week; one per week; 2–4 times per week; 5–6 times per week; once or more daily; unknown)
➢ Cereal consumption (bowls per week)
➢ Cheese consumption (never; < one per week; one per week; 2–4 times per week; 5–6 times per week; once or more daily; unknown)
➢ Salt added to food (never/rarely; sometimes; usually; always; unknown)
➢ Type of milk used (never/rarely; other types; soya; skimmed; semi-skimmed; full cream; unknown)
➢ Fish consumption (never; < one per week; one per week; 2–4 times per week; 5–6 times per week; once or more daily; unknown)
➢ Sunscreen usage (never/rarely; sometimes; usually; always; unknown)
➢ Ease of skin tanning (very tanned; moderately tanned; mildly/occasionally tanned; never tan/only burn; unknown)
➢ Job exposure to hazardous materials (none; rarely; sometimes; often; unknown)
➢ Aspirin prescribed (no; yes)
➢ Warfarin prescribed (no; yes)
➢ Digoxin prescribed (no; yes)
➢ Metformin prescribed (no; yes)
➢ Oral contraceptives prescribed (no; yes)
➢ Hormone replacement therapy prescribed (no; yes)
➢ Anti-hypertensive drugs prescribed (no; yes)
➢ Statins prescribed (no; yes)
➢ Previously diagnosed with *h*. *pylori* infection (no; yes)
➢ Previously had radiotherapy (no; yes)
➢ Previously diagnosed with bowel polyps (no; yes)
➢ Previously diagnosed with Coeliac disease (no; yes)
➢ Previously diagnosed with Crohn’s disease (no; yes)
➢ Previously diagnosed with thyroid disease (no; yes)
➢ Previously diagnosed with acid reflex (no; yes)
➢ Previously diagnosed with hyperplasia (no; yes)
➢ Previously diagnosed with prostate disease (no; yes)
➢ Previously diagnosed with cancer (no; yes)
➢ Previously diagnosed with coronary heart disease [CHD] (no; yes)
➢ Previously diagnosed with stroke/transient ischemic attack [TIA] (no; yes)
➢ Previously diagnosed with Type II diabetes [T2DM] (no; yes)
➢ Previously diagnosed with chronic obstructive pulmonary disease [COPD] (no; yes)

### Outcome

The primary outcome was all-cause mortality registered at the UK Office of National Statistics (ONS). All deaths in the UK are registered with the ONS which provides a gold-standard outcome. The primary underlying cause of death was determined by the International Classification of Diseases 10^th^ Edition (ICD-10).

### Study design

One researcher (SW) aimed to develop prognostic algorithms based on supervised learning to predict a binary outcome for death, using two machine-learning approaches (deep learning using a neural network [14] and random forest [15]). Another researcher (LV) used a traditional model building process from survival models using multivariate Cox regression. Prognostic algorithms are generally functions that map independent variables to a value between zero and one that corresponds to the risk of the dependent variable occurring. Machine-learning algorithms aim to learn the function from the training dataset input variables (features). Many machine-learning algorithms have hyper-parameters that define a specific function (with parameters) to be learned. For example, a random forest algorithm [15] has hyper-parameters specifying the number of trees and the max depth of each tree (effectively how many interactions are considered in the model), whereas the decision rules are the parameters. A neural network [14] has hyper parameters which control for the complexity of the model, size of the network, and how network connections are activated, or essentially “learned”. We elected to evaluate random forest and deep learning models to demonstrate opposite ends of the spectrum in terms of complexity. Random forest are one simplest (minimum hyper-parameters) and most interpretable algorithms to train, validate and interpret whereas deep learning models are more complicated to develop and interpret, with a large number of hyper-parameters to tune.

A process known as cross-validation is often implemented to tune the hyper-parameters while trying to reduce the occurrence of overfitting. Once the optimal hyper-parameters have been tuned via cross-validation on the training dataset, the final model parameters are then determined on the whole training dataset and the model is applied to the test set to estimate the performance on new data.

To develop these prediction algorithms, the cohort was randomly partitioned into a “training” cohort in which all cause-mortality algorithms were derived and a “test” cohort in which the algorithms could be applied and their accuracy assessed. The training cohort was derived from random sampling of 75% (n = 376,971) of the cohort; and the test cohort, comprised of the remaining 25% (n = 125,657), was set aside until the final algorithms were developed and could be applied to make predictions.

### Deep learning

**Fig 1:**
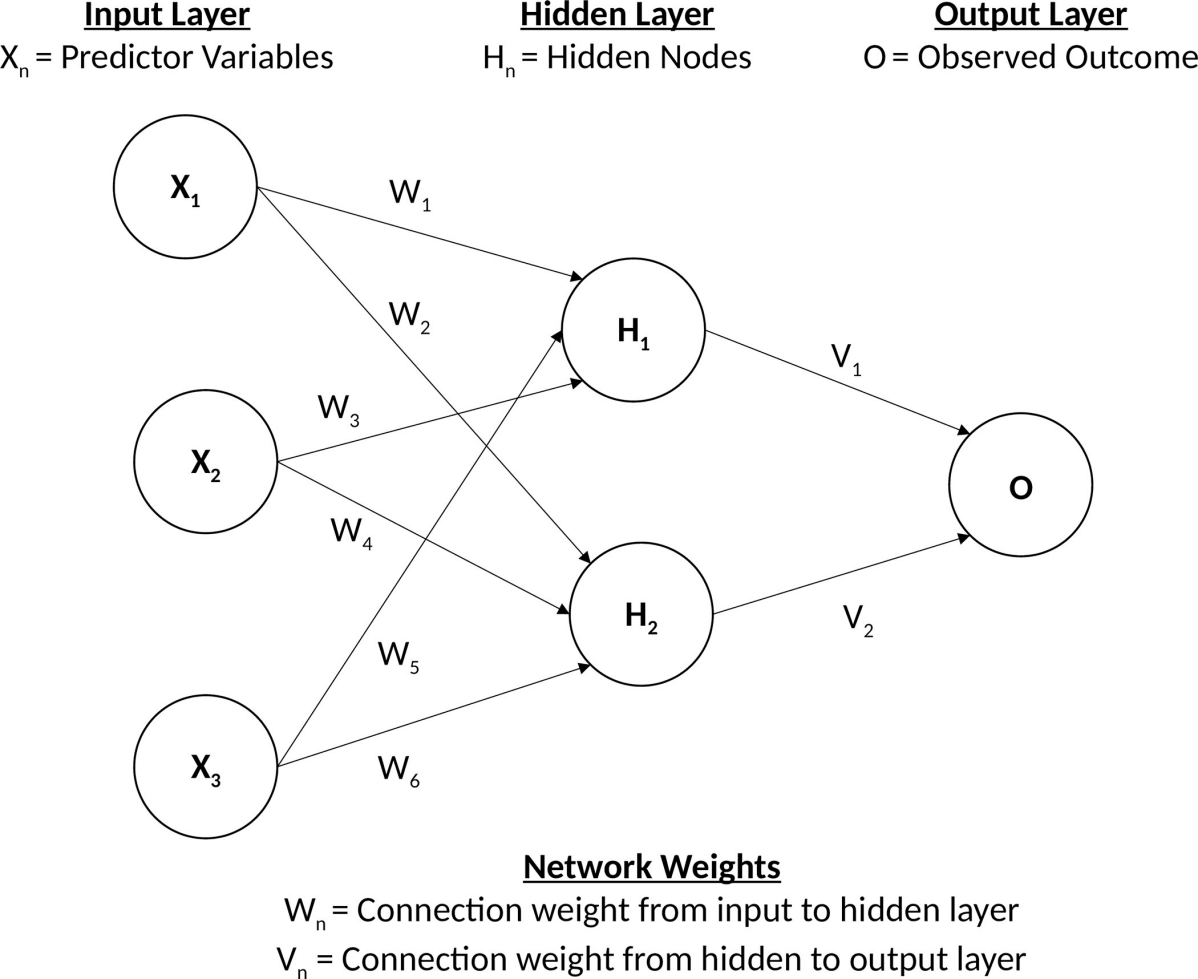
Example of a single hidden layer feed forward neural network.

We first developed a deep learning algorithm based on a multi-layer feedforward artificial neural network (ANN) that was trained with a stochastic gradient descent using back-propagation [16]. Gradient descent using back-propagation is an optimisation algorithm for finding the local minimum of the loss function based on calculating errors between predicted and observed outputs, and iteratively moving down a “gradient” to identify the most accurate predictions which reduce classification error. **Fig 1** illustrates a single hidden layer feed-forward neural network consistent of a single input layer **X**_**n**_, single hidden layer consisting of **H**_**n**_ nodes, and output layer **O**, representing a classification outcome. The output of each non-input layer is the weighted combination of the previous layers nodes outputs transformed by a pre-defined non-linear function such that **H**_**n**_ **= f (∑ W**_**n**_ **)** and O_n_ = **f (∑ H**_**n**_ **V**_**n**_**).**

This network based on deep learning can contain a large number of hidden layers consisting of neurons with *tanh*, *rectifier*, *and maxout* non-linear activation functions. Advanced features such as adaptive learning rate, rate annealing, momentum training, drop out, *L1* and *L2* regularisation, check-pointing, and grid-search enable high predictive accuracy. Full descriptions of all hyper-parameters and explanation of their functions can be found elsewhere (http://docs.h2o.ai/h2o/latest-stable/h2o-docs/data-science/deep-learning.html).

To determine the optimal hyper-parameters for the neural network, a grid-search was conducted (in epidemiological terms–a large-scale sensitivity analysis) using 10-fold cross-validation (where the training cohort of 376,971 participants was partitioned into nine subsets for training and one subset for validation). A grid search was conducted where we sampled uniformly from the set of all possible hyper-parameter value combinations listed in **Box 2**:

**Box 2. Hyper-parameter list used to conduct grid-search to optimise deep learning neural network**

Hidden layers = 1–5

Nodes in each layer = 5–100 in increments of 5

Epochs = 1–200

Fold-assignment = Stratified

Activation = *Rectifier*, *Maxout*, *Tanh*, *RectifierWithDropout*, *MaxoutWithDropout*, *TanhwithDropout*

Rho = 0.9, 0.95, 0.99, 0.999

Epsilon = 1e-10, 1e-8, 1e-6, 1e-4

Input drop-out ratio = 0, 0.1, 0.2

Rate = 0, 0.01, 0.005, 0.001

Rate annealing = 1e-8, 1e-7, 1e-6

Momentum = 0.5

Stopping rounds = 0

L1 = 0, 0.00001, 0.0001

L2 = 0, 0.00001. 0.0001

Max weight = 10, 100, 100, 10e38

The optimal hyper-parameters were determined by maximising the area under the receiver operating characteristic curve (AUC) through 10-fold cross-validation because the AUC is less affected when classification data are unbalanced (UK Biobank cohort has more people who are alive at follow-up than those who died). Prediction made by the deep learning algorithm gave the probability of either being alive at the end of follow-up or an individual dying within up to 10 years of follow-up, with the sum of these probabilities equal to one. The deep learning algorithm was developed using *RStudio* with the library package *h2o* (http://www.h2o.ai).

### Random forest

The second prediction algorithm we developed was based on a random forest decision tree [15], which averages multiple decision tree predictions. The algorithm involves learning *ntree* diverse decision trees *f_i_*(*x*):*X* → *Y* where **X** is the input variables and Y is the outcome, each trained using a different random selection (with replacement) of *mtry* variables. The overall prediction then takes the majority voting of all *ntree* decision trees, described below:

$$(x)=\left\{ \begin{array}{l} 1,\;if\frac{\sum_{i}f_{i}(x)}{ntree}>0.5\\ 0,\;otherwise \end{array}\right.$$

The hyper-parameters of the random forest controls the complexity of the learning function. A random forest with a high max depth (maximum number of interactions between independent variables) and high *mtry* (number of variables included in each tree) is more complex and thus, more likely to cause over-fitting. Therefore, limiting the max depth or *mtry* can effectively perform regularisation and reduce the chance of overfitting. This can also be accomplished by sampling the number of data points to be used for each tree (row sample). For the hyper-parameter grid search we investigated the following parameters (**Box 3**):

**Box 3. Hyper-parameter list used to conduct grid-search to optimise random forest algorithm**

Ntree = 50, 100, 500

Mtry = 5–60 in increments of 5

Max depth = 2,4,6,8,10

Row sample = 90%, 95%, 100%

Similar to deep learning, we used 10-fold cross validation within the training cohort to maximise the AUC. Predictions made by the random forest algorithm gave the probability of an either being alive and of dying at the end of follow-up, with the sum of these probabilities equal to one. The random forest algorithm was developed using *RStudio* with library package *caret* (http://CRAN.R-project.org/package=caret).

### Survival modelling

The final prediction algorithms were developed from traditional risk modelling approaches developing survival models using Cox regression. To develop the algorithms, we first assessed association between all baseline variables and death. All variables with p-value less than 10% were then included in a multivariate Cox regression model. Then a systematic backwards elimination method was performed on the basis of their p-value, from maximum to minimum. Simultaneous predictions were made for each model on the training cohort in an iterative process to evaluate the loss of performance based on the AUC. The process was repeated until a significant loss of performance occurred. The optimal regression model was identified as the model with the least number of variables without a significant decrease in model performance, ensuring the model did not over-fit while maintaining predictive accuracy. To have a baseline simple model for comparison, we also created a Cox model using baseline age and gender, as age is likely to be the primary risk factor for death. All models were tested for proportional hazards assumptions and inclusion of fractional polynomials. The models were then used to derive a survival risk function to predict the probability of dying during a 10-year follow-up period:

$$10\;year\;probability\;of\;death=1-S^{\exp\left(A-B\right)}$$

**S** is the 10-year survival rate, **A** is the linear prediction function derived from multivariate Cox regression, and **B** is the mean prediction constant of the linear prediction function derived from Cox regression. The algorithm based on Cox models were developed using *STATA 15 SE*.

### Statistical analysis

Descriptive characteristics of the study population were provided, including number (%) and mean (SD) for categorical and continuous variables, respectively. Multiple imputation using chained equations [17] derived 10 copies of the original data to impute missing values which were missing-at-random. Little’s test for missing completely-at-random [18] was used, which performs a likelihood-ratio test with adjustment for equal variances. All continuous variables were found to be missing completely at random (p > 0.05), and thus multiple imputation was performed. Categorical variables contained an “unknown” response category, which participants could select which we did not view as this missing at random and coded this response separately.

To compare deep learning and random forest algorithms with survival models, predictions were made on 10-year all-cause mortality in the test cohort (n = 125,657). An additional analysis was conducted by re-training the models to predict vascular (coronary heart disease/cerebrovascular disease) and non-vascular causes separately, and compare their performance. The performance of the prediction algorithms was assessed by discrimination (calculating Harrell’s *c*-statistic, which corresponds to the AUC) [19]. This gives the probability that a randomly selected participant who died during follow-up has a higher risk of mortality than a participant who was alive at the end of follow-up, ranging from 0.5 (pure chance) to one (perfect discrimination). Standard errors and 95% confidence intervals were estimated for the *c*-statistic using a jack-knife procedure [20].

Calibration, defined by how closely the predicted risk of mortality agrees with the expected risk of mortality, was assessed by plotting predicted risk against the observed risk. We also conducted binary classification analysis to determine an optimum point where risk of mortality could be considered “high” and “normal”, comparing each risk algorithm by calculating sensitivity and specificity

## Results

### Study population

After three participants were excluded from the analysis as their death date was recorded before their start date (likely due to clerical error), there were a total of 502,625 participants in the entire cohort who were followed up for a total of 3,508,454 person-years, resulting in 14,418 deaths (2.9%). **Table 1** describes in detail the baseline characteristics of the study population. There were significantly more men than women (60.7% M, 39.3% F) who died during follow-up, whereas more women than men (54.8% F, 45.5% M) were alive at the end of follow-up. The mean baseline age of those who had died (61.3 years) was also significantly greater than those who were alive (56.4 years) at the end of follow-up. Those who died were also significantly less likely to be of White ethnicity, had less education/qualifications, more history of chronic conditions, less healthy in terms of biometrics and lifestyle choices, and were from more deprived backgrounds.

**Table 1:** Selected baseline characteristics of the study population aged 40–69 years recruited between 2006 to 2010 stratified by mortality during follow-up. *Categorical variables are numbers and proportions and continuous variables are mean and standard deviations*.

Characteristics	Alive (n = 488,207)	Died (n = 14,418)	p-value
**Gender**			
Female (%)	267,792 (54.8)	5,667 (39.3)	—
Male (%)	220,415 (45.5)	8,751 (60.7)	< 0.001
**Baseline age [years] (SD)**	56.4 (8.1)	61.3 (6.6)	< 0.001
**Ethnicity**			
White (%)	458,948 (94.0)	13,860 (96.1)	—
South Asian (%)	9,712 (2.0)	170 (1.2)	< 0.001
East Asian (%)	1,552 (0.3)	22 (0.2)	0.003
Black (%)	7,951 (1.6)	114 (0.8)	< 0.001
Other/mixed (%)	7,371 (1.5)	147 (1.0)	< 0.001
Unknown (%)	2,673 (0.6)	105 (0.7	0.008
**Qualifications**			
None (%)	80,930 (16.6)	4,361 (30.3)	—
College/University (%)	157,904 (32.3)	3,298 (22.9)	< 0.001
A/AS Levels (%)	54,076 (11.1)	1,257 (8.7)	< 0.001
O Levels/GCSEs (%)	102,535 (21.0)	2,683 (18.6)	< 0.001
CSEs (%)	26,370 (5.4)	523 (3.6)	< 0.001
NVQ/HND/HNC (%)	31,671 (6.5)	1,065 (7.4)	< 0.001
Other Professional Qualifications (%)	25,009 (5.1)	801 (5.5)	< 0.001
Unknown (%)	9,712 (2.0)	430 (3.0)	< 0.001
**Smoking**			
Non-smoker (%)	438,137 (89.7)	11,502 (79.8)	—
Current smoker (%)	50,070 (10.3)	2,916 (20.2)	< 0.001
**Previously diagnosed with T2DM**			
No (%)	466,947 (95.6)	12,721 (88.2)	—
Yes (%)	21,260 (4.4)	1,697 (11.8)	< 0.001
**Previously diagnosed with cancer**			
No (%)	446,616 (91.5)	11,181 (77.6)	—
Yes (%)	41,591 (8.5)	3,237 (22.4)	< 0.001
**Previously diagnosed with CHD**			
No (%)	469,221 (96.1)	12,666 (87.9)	—
Yes (%)	18,986 (3.9)	1,752 (12.1)	< 0.001
**Previously diagnosed with stroke/TIA**			
No (%)	481,876 (98.7)	13,798 (95.7)	—
Yes (%)	6,331 (1.3)	620 (4.3)	< 0.001
**Previously diagnosed with COPD**			
No (%)	482,355 (98.8)	13,761 (95.4)	—
Yes (%)	5,852 (1.2)	657 (4.6)	< 0.001
*Cigarettes per day (SD)	1.5 (5.0)	3.5 (8.0)	< 0.001
*Waist circumference [cm] (SD)	90.2 (13.4)	95.1 (14.7)	< 0.001
*Height [m] (SD)	168.4 (9.3)	169.3 (9.1)	< 0.001
*Weight [kg] (SD)	77.9 (15.9)	80.9 (17.7)	< 0.001
*Body fat percentage [%] (SD)	31.4 (8.5)	30.9 (8.5)	< 0.001
*Body mass index [kg/m^2] (SD)	27.4 (4.8)	28.1 (5.4)	< 0.001
*Systolic blood pressure [mm HG] (SD)	139.5 (19.1)	143.6 (20.3)	< 0.001
*Diastolic blood pressure [mm HG] (SD)	82.2 (10.4)	82.2 (10.9)	0.998
*Physical activity [MET-min per week] (SD)	1915.1 (2856.2)	1704.5 (2840.4)	< 0.001
*Forced expiratory volume 1 [L] (SD)	2.8 (0.8)	2.6 (0.8)	< 0.001
*Townsend deprivation index (SD)	-1.3 (3.1)	-0.6 (3.4)	< 0.001

* Missing values: Weight: 0.55% missing; Height: 0.50% missing; BMI: 0.62% missing; Waist circumference: 0.43% missing; Body fat percentage: 2.08% missing; Diastolic blood pressure: 6.92% missing; Systolic blood pressure: 6.93% missing; FEV1: 9.71% missing; Cigarettes per day: 3.35% missing; Townsend index: 0.13% missing

The primary underlying cause of death was most commonly due to cancers (n = 9,477; 65.7%), particularly cancers of the digestive organs (n = 2,775; 19.2%) and cancers of the respiratory organs (n = 1,832; 12.7%). The second most common underlying cause of death was from disease of the circulatory system (n = 3,438; 23.8%), with the most common being death from coronary heart disease (n = 1,931; 13.4%) and cerebrovascular disease (n = 623; 4.3%). Diseases of the respiratory system (most commonly COPD), were responsible for 943 deaths (6.5%).

### Generalisability of the UK Biobank cohort

**Fig 2:**
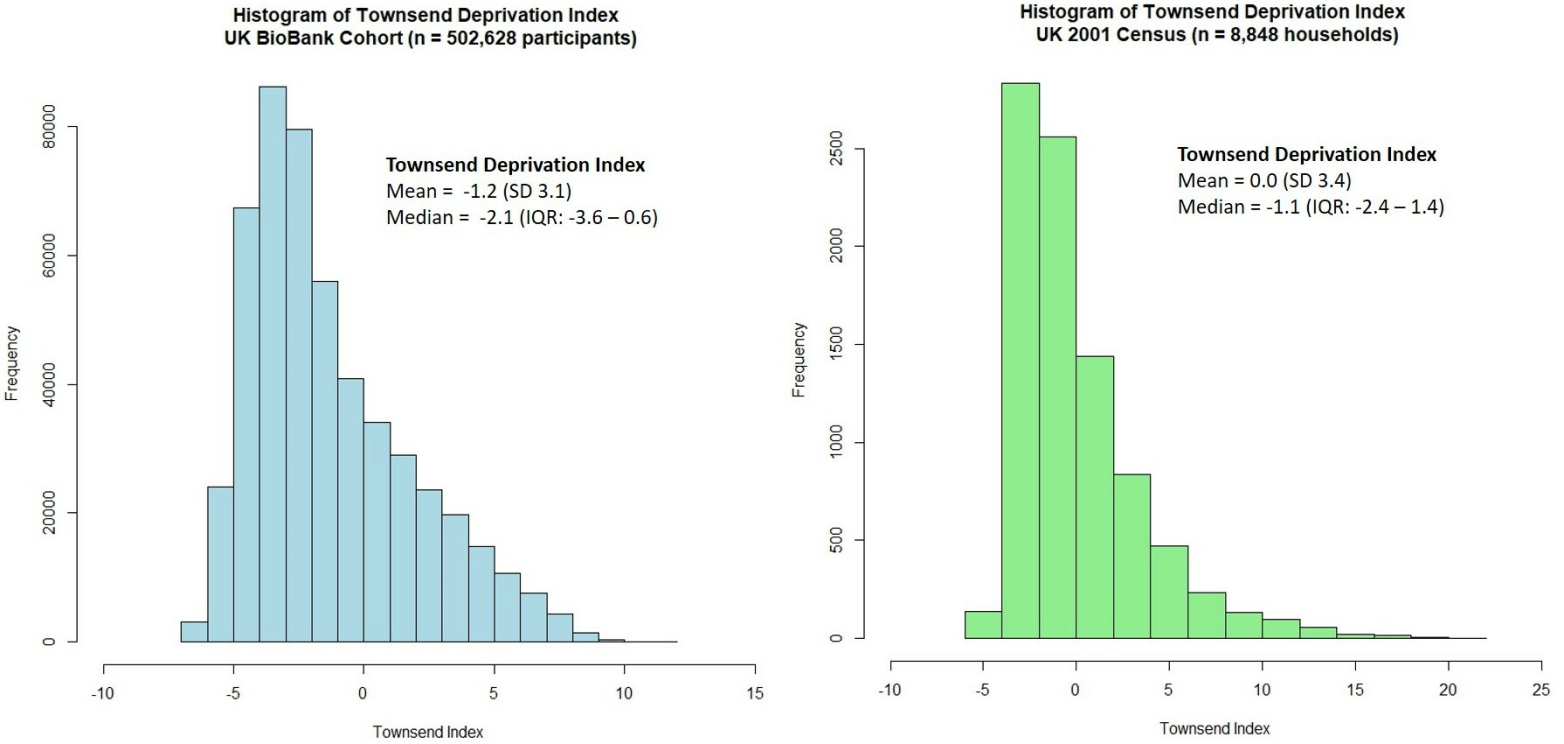
Comparing townsend deprivation index between UK Biobank participants and households in the 2001 UK Census.

As 32.7% of the UK Biobank cohort had a College/University degree at baseline, we investigated how the cohort compared to the general UK population. The Townsend deprivation index is an area level measure of material deprivation [21] with higher levels indicating more deprived areas. Using UK 2001 census data [22], we compared the Townsend deprivation index gathered from 8,848 households with that of the 502,625 participants in the UK Biobank cohort (**Fig 2**). Compared to the UK census data, individuals recruited into the UK Biobank were generally less deprived (median Townsend score = -2.14, Interquartile range: -3.64 to 0.55) compared to the general UK population (median Townsend score = -1.05, Interquartile range: -2.40 to 1.44).

### Mortality prediction algorithms

The optimal neural network algorithm identified from deep learning by conducting a grid-search using 10-fold cross-validation in the training cohort (n = 376,971) had the following hyper-parameters: 3 hidden layers with 50 nodes in each layer, 1.51 epochs, stratified fold assignment, activation = *tanh*, *rho* = 0.999, *epsilon* = 1E-10, input dropout ratio = 0.2, momentum = 0.5, rate = 0.001, rate annealing = 1E-07, stopping rounds = 0, *L1* = 1E-05, *L2* = 1E-04, Max weight = 1000. Similarly, the optimal random forest algorithm identified from a grid-search in the training cohort (n = 376,971) also using 10-fold cross validation was found to have hyper-parameters *ntree =* 200, *mtry =* 35, *max depth =* 3, row sample = 95%. These hyper-parameters control the machine-learning process and allow replication of the algorithms. The final adjusted Cox regression model is shown in **Table 2**.

**Table 2:** Adjusted hazard ratios from final multivariable Cox regression model predicting 10-year mortality in the training cohort (n = 376,971).

Predictor Variables	Hazard Ratio	P-Value	95% Confidence Interval	
			Lower Confidence Limit	Upper Confidence Limit
**Gender**				
Female	Ref	—	—	—
Male	2.17	< 0.001	2.08	2.27
**Log age**	44.00	< 0.001	36.35	53.27
**Qualifications**				
None	Ref	—	—	—
College/University	0.75	< 0.001	0.71	0.80
A/AS Levels	0.83	< 0.001	0.77	0.89
O Levels/GCSEs	0.81	< 0.001	0.76	0.85
CSEs	0.88	0.020	0.79	0.98
NVQ/HND/HNC	0.80	< 0.001	0.74	0.87
Other professional qualifications	0.79	< 0.001	0.73	0.87
Unknown	0.97	0.590	0.86	1.09
**Ethnicity**				
White	Ref	—	—	—
South Asian	0.59	< 0.001	0.49	0.70
East Asian	0.67	0.110	0.41	1.09
Black	0.63	< 0.001	0.51	0.78
Other/Mixed	0.81	< 0.030	0.66	0.98
Unknown	1.00	0.990	0.78	1.27
**Previous diagnosis of cancer**				
No	Ref	—	—	—
Yes	2.58	< 0.001	2.47	2.71
**Previous diagnosis of CHD**				
No	Ref	—	—	—
Yes	1.58	< 0.001	1.49	1.68
**Previous diagnoses of T2DM**				
No	Ref	—	—	—
Yes	1.72	< 0.001	1.62	1.83
**Previous diagnosis of COPD**				
No	Ref	—	—	—
Yes	1.87	< 0.001	1.71	2.05
**Smoking**				
Non-smoker	Ref	—	—	—
Current smoker	2.01	< 0.001	1.91	2.11
**Log diastolic blood pressure**	0.73	< 0.001	0.60	0.89
**Log systolic blood pressure**	1.14	0.200	0.94	1.38
**Log Townsend deprivation index**	1.13	< 0.001	1.10	1.17
**Log body mass index**	1.12	0.070	0.99	1.27
**Log MET-min per week**	0.94	< 0.001	0.93	0.95
**Log forced expiratory volume 1**	0.53	< 0.001	0.51	0.56

The final model was reduced down to 15 predictor variables which maintained the maximum discrimination through stepwise model building and backwards variable elimination. The model included biometric variables (diastolic and systolic blood pressure, BMI and FEV1), smoking, physical activity, age, gender, ethnicity, educational qualifications, Townsend deprivation index, and four variables of previous medical history of common chronic conditions (cancer, CHD, T2DM and COPD). Most variables retained statistical significance at the 5% level.

Expectedly, log age was the strongest predictor of mortality (HR = 44.95, 95% CI 36.35–53.27) in the final model. In a simple prediction model based on only log age and adjusting for gender, being male significantly increased risk of mortality (HR = 1.73, 95% CI 1.62–1.85) and log age demonstrated an even stronger association with mortality (HR = 131.60, 95% CI 98.30–176.16).

### Variable importance

Comparing the Cox model to random forest and deep learning showed overlap in the top contributing risk factor variables for predicting death **(Table 3**). Six of the 15 top rank risk factors (age, prior diagnosis of cancer, gender, smoking, FEV1, education) were identified in all three algorithms. The Cox model overlapped with the either the random forest or the deep learning algorithm for seven risk factors (prior diagnosis of COPD, prior diagnosis of T2DM, prior diagnosis of CHD, diastolic and systolic blood pressure, BMI, Townsend deprivation index). Ethnicity and physical activity (represented by MET-min per week) were important predictors in the Cox model but were not identified as important in the random forest and deep learning models. Instead, the random forest model put emphasis on other measures of adiposity including waist circumference, body fat percentage, and interestingly included skin tone, and two measures of healthy diet (vegetable and fruit consumption). The deep learning model identified alcohol consumption, medication prescribing (digoxin, warfarin, statins), and environmental factors such as residential air pollution and job related hazardous exposures. Full variable importance rankings for all the variables from ML can be found in **S1 Table** for random forest and **S2 Table** for deep learning.

**Table 3:** Top 15 risk factor variables for predicting mortality listed in descending order of “importance” by algorithm derived from the training cohort of 376,971 patients.

Cox model ^a^	Random Forest ^b^	Deep Learning ^c^
Age	BMI	Smoking
Prior diagnosis of cancer	FEV1	Age
Gender	Waist circumference	Prior diagnosis of cancer
Smoking	Diastolic blood pressure	Alcohol consumption
Prior diagnosis of COPD	Systolic blood pressure	Digoxin prescribed
FEV1	Age	Gender
Prior diagnosis of T2DM	Body fat percentage	Warfarin prescribed
Prior diagnosis of CHD	Smoking	Townsend deprivation index
Diastolic blood pressure	Prior diagnosis cancer	Residential air pollution
BMI	Gender	Prior diagnosis of CHD
Systolic blood pressure	Skin tone	Statins prescribed
Townsend deprivation index	Education	Prior diagnosis of COPD
Ethnicity	Prior diagnosis T2DM	Job exposure to hazardous materials
MET-min week	Vegetable consumption	Education
Education	Fruit consumption	FEV1

^a^ ranking determined by strongest to weakest Cox regression coefficients

^b^ ranking determined by largest to smallest mean decreases in accuracy

^c^ ranking determined by largest to smallest scaled importance derived from network weights

orange = top risk factor in all three algorithms; blue = top risk factor in two algorithms; green = top risk factor in one algorithm

### Prediction accuracy

**Fig 3:**
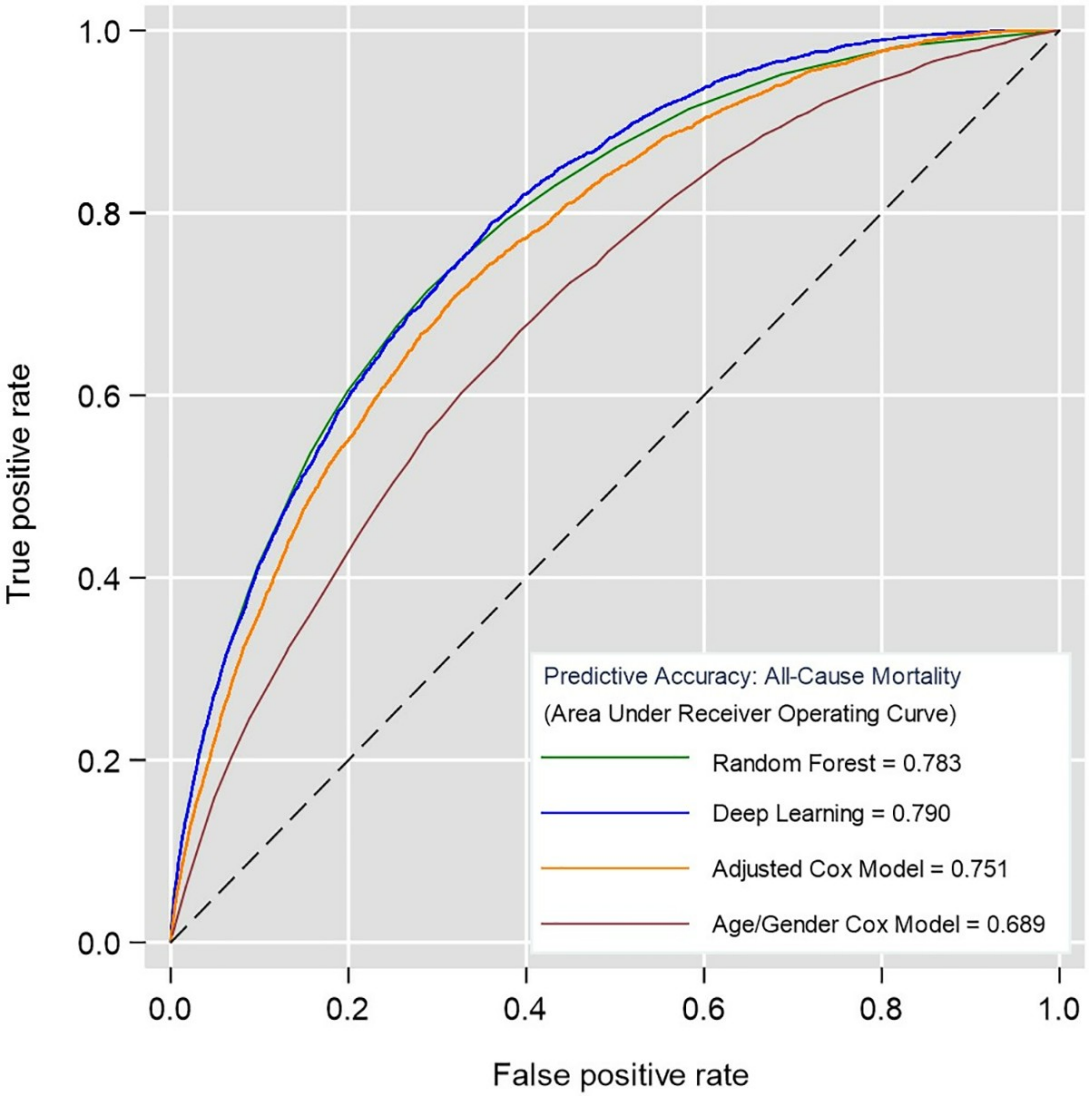
Receiver operating curves derived from predicting all-cause mortality in the test cohort (n = 125,657) using Cox models, random forest and deep learning. Higher area under the curve (c-statistic) shows better discrimination.

The algorithms were applied to the test cohort (n = 125,657) to predict each individual’s risk of all-cause mortality. The corresponding discrimination (AUC *c-*statistic) of each algorithm is shown in **Fig 3**.

The simple age and gender Cox regression model served as a baseline for comparison (AUC 0.689, 95% CI 0.681–0.699). The fully adjusted Cox regression model presented in **Table 2** significantly improved discrimination by 6.2% (AUC 0.751, 95% CI 0.748–0.767). The application of machine-learning algorithms further improved discrimination by 3.2% using random forest (AUC 0.783, 95% CI 0.776–0.791) and 3.9% using deep learning (AUC 0.790, 95% CI 0.783–0.797). These ML algorithms improved discrimination by 9.4% and 10.1% respectively, from a simple age and gender Cox regression model. Random forest and deep learning achieved similar levels of discrimination with no significant difference.

Improvements in model performance stratified by vascular and non-vascular causes of death in the validation cohort demonstrated comparable increases in discrimination comparing ML algorithms to Cox models (**S3 Table**). Both Random forest and deep learning models improved discrimination for predicting vascular causes of death by 2.7% and 10.5% compared to the fully adjusted and simple age/gender Cox models, respectively. For predicting non-vascular causes of death, random forests improved discrimination by 2.2% and 8.7% compared to the fully adjusted and simple age/gender Cox models. The deep learning model improved discrimination by 3.1% and 9.6% compared to the fully adjusted and simple age/gender Cox models. As a whole, the models had much higher accuracy in predicting vascular causes of death compared to non-vascular causes. For instance, the deep learning algorithm resulted in an AUC of 0.864 (95% CI 0.854–0.873) for predicting vascular causes of death compared to 0.755 (95% CI 0.747–0.763) for predicting non-vascular causes of death.

### Calibration accuracy

**Fig 4:**
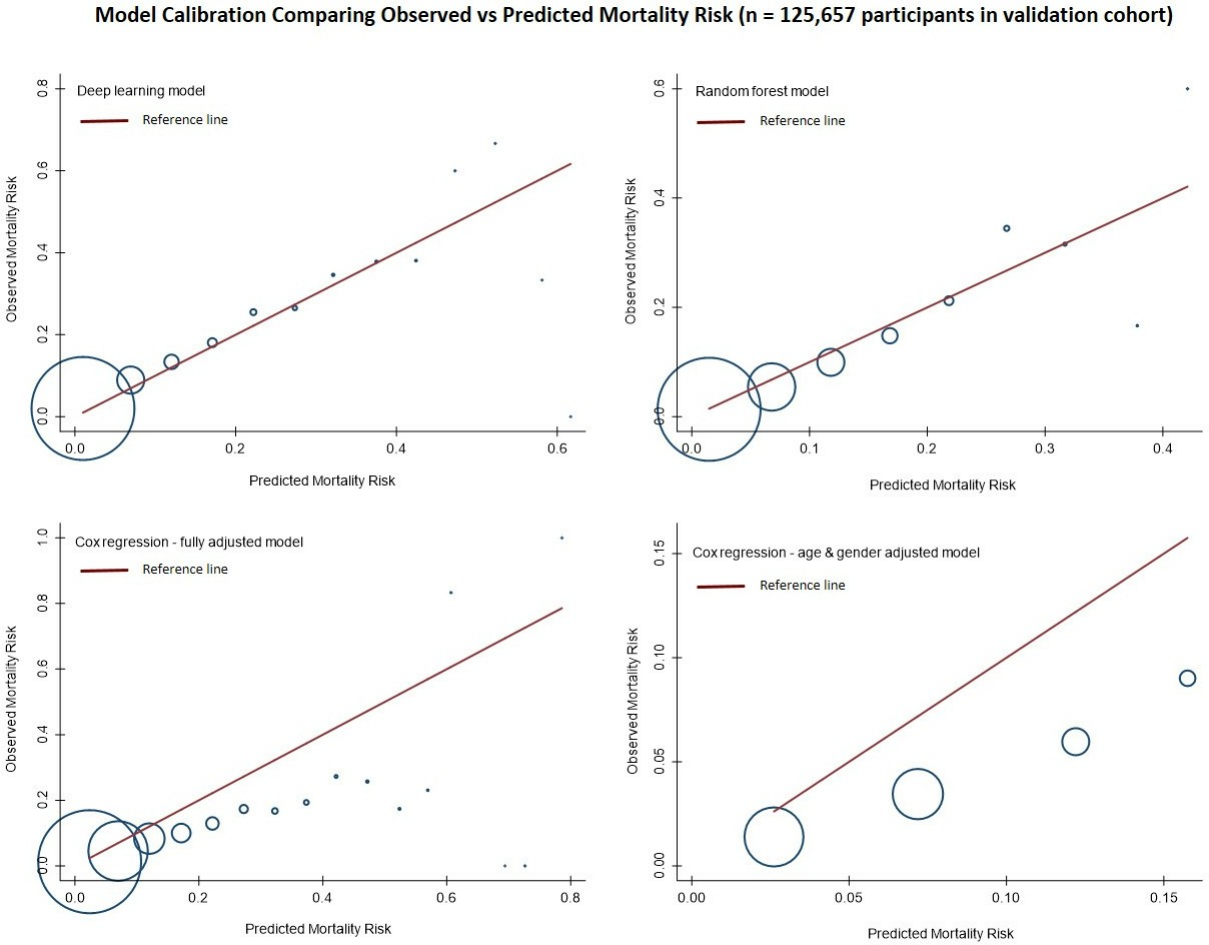
Calibration of algorithms for predicting all-cause mortality in the test cohort (n = 125,657). Red line indicates predicted risk of mortality plotted against observed proportion of mortality in blue circles across deciles of predicted risks.

Calibration of the algorithms is presented in **Fig 4** by plotting deciles of predicted risk against expected proportion mortality in each decile. Deep learning was found to have the best calibration with the predicted risks mapping to expected proportion of mortality. The random forest algorithm was calibrated well at lower predicted risks but not as well-calibrated at higher predicted risks. Both Cox regression models over-predicted risks across all deciles.

### Classification analysis

**Fig 5:**
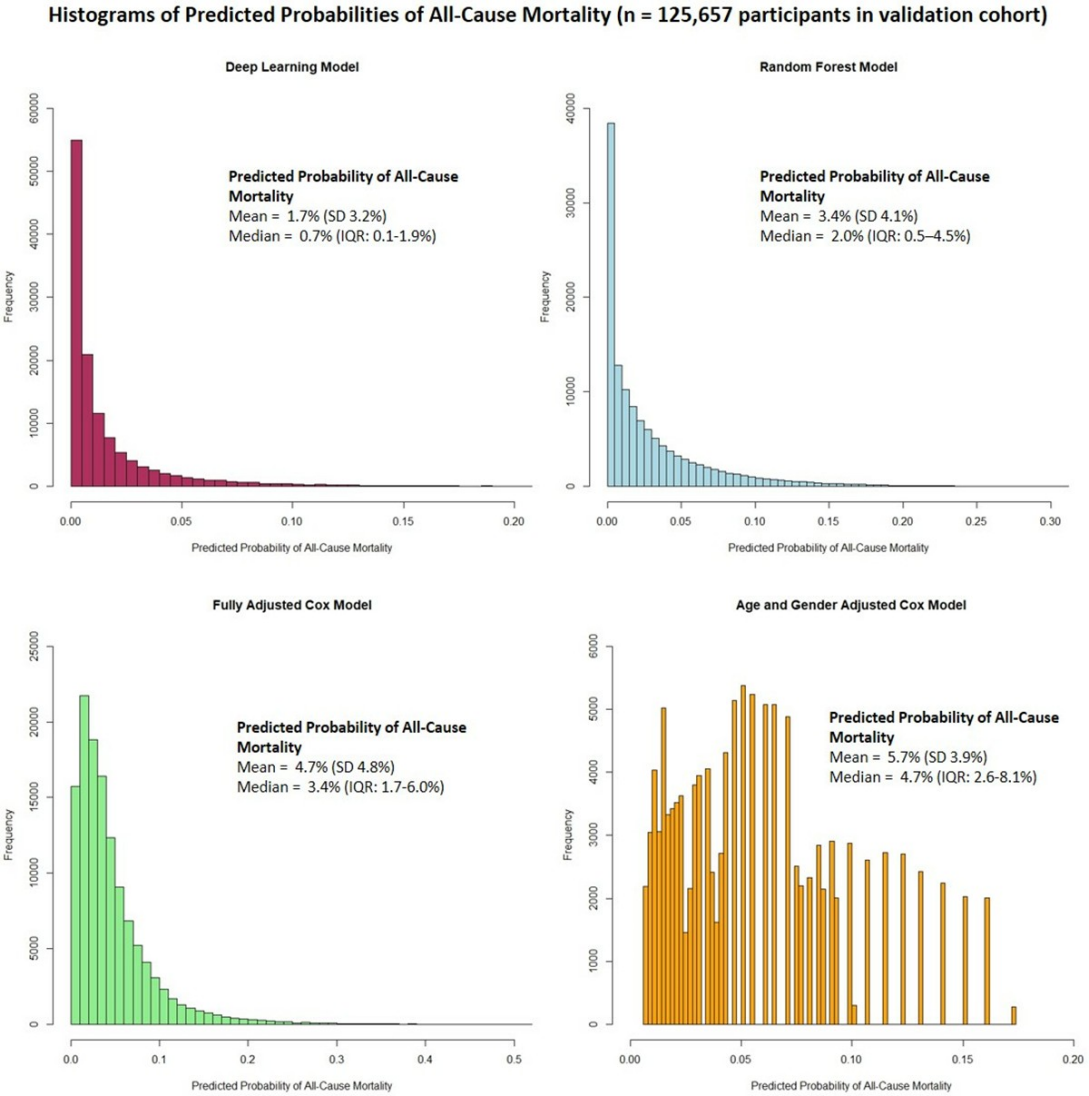
Distribution of predicted risks for all-cause mortality in the test cohort (n = 125,657).

For binary classification analysis, an optimal threshold for determining “high” and “normal” risk of mortality was created by standardising the threshold based on the predicted distributions of risks for each of the algorithms shown in **Fig 5**.

Since the deep learning algorithm had highest discrimination and was well-calibrated, we determined an optimal threshold where sensitivity and specificity intersected to maximise the overall AUC from a binary classification. This was determined as > 2% for the deep learning algorithm to determine “high” risk of mortality. To allow for direct comparison, the corresponding threshold was determined for each algorithm, taking into account the different distributions. The results of the classification analysis are shown in **Table 4**.

**Table 4:** Binary classification accuracy comparing each algorithm for predicting “high” risk of mortality in the test cohort (n = 125,657).

Algorithm	Optimal Threshold	Correctly Classified Death	Correctly Classified Alive	Sensitivity	Specificity
Deep Learning	> 2%	2,343/3,608	92,978/122,049	64.9%	76.2%
Random Forest	> 5%	2,300/3,608	94,603/122,049	63.7%	77.5%
Adjusted Cox Model	> 6%	2,197/3,608	92,832/122,049	60.9%	76.1%
Age/Gender Cox Model	> 8.4%	1,728/3,608	93,661/122,049	43.7%	76.7%

The deep learning algorithm predicted 2,343 individuals correctly who had died during follow-up, resulting in the highest sensitivity (76.2%). Compared to deep learning, the random forest algorithm predicted 43 fewer individuals who had died, resulting in a slight decrease in sensitivity to 63.7%. However, the random forest algorithm did improve prediction of 1,625 individuals who were alive, resulting in the highest specificity of all algorithms (77.5%).

Compared to deep learning (algorithm with highest sensitivity), both algorithms based on Cox regression predicted fewer individuals who had died (n = 146 for the adjusted Cox model; n = 615 for age/gender Cox model). Compared to random forest (algorithm with the highest specificity), both algorithms based on Cox regression also predicted fewer individuals who were alive (n = 1,771 for the adjusted Cox model; n = 942 for the age/gender Cox model). The age/gender Cox model also showed a sharp drop-off in sensitivity to 43.7% compared to the other algorithms but retained specificity comparable to the other algorithms.

## Discussion

### Principal findings

We found that machine-learning algorithms were better at predicting individuals who died prematurely, with higher discrimination, better calibration and classification accuracy, when compared to standard approaches. Deep learning based on a multi-layer feed-forward artificial neural network performed the best, with predictive accuracy improving by 3.9% compared to a multivariate Cox regression model, and by 10.1% using just age and gender alone. ML algorithms, when predicting vascular and non-vascular causes of death separately, demonstrated similar improvements in predictive accuracy.

The study shows the value of using ML, to explore a wide array of individual clinical, demographic, lifestyle and environmental risk factors, to produce a novel and holistic model that was not possible to achieve using standard approaches. This work suggests that use of ML should be more routinely considered when developing models for prognosis or diagnosis.

### Comparison with existing literature

Current research using ML approaches has generally fallen into two categories: for the development of diagnostic applications [3, 4, 7], and for prognosis research using observational data (i.e. risk prediction modelling) [5,6]. For diagnostic applications where ML algorithms are trained using images, notable studies include using neural networks to diagnose skin cancer, with accuracy on a par with 21 expert dermatologists [3] or detecting diabetic retinopathy with near perfect accuracy (AUC of 0.99) [4]. Others have utilised ML techniques based on decision trees and support vector machines to predict non-small cell lung cancer with high levels of accuracy [7].

For prognostic research or risk prediction modelling, examples include applying ML algorithms to predict psychosis using clinical trial data [6], and our cohort study using the routine clinical data of nearly 400,000 patients in primary care which demonstrated ML algorithms significantly improve prediction of future cardiovascular disease compared to standard clinical guidelines [5]. The current study, using the UK Biobank cohort of over 500,000 participants, is larger than all previous studies using ML in either diagnostic or prognosis research.

While all these studies demonstrate the considerable promise of ML, lack of reproducibility is a potential concern. For example, in the diabetic retinopathy study noted above, a separate replication study achieved a much lower AUC of 0.74 [23]. This may not only be due to the nature of the methodology, but also to lack of reporting on specific model parameters and structure. This presents a unique challenge for ML algorithms as adoption into clinical practice will and should require formal replication and validation.

### Strengths

To our knowledge, this is the first investigation of this type using ML. In sample size, recentness, and capture of biometrics and demographic variables, including a diverse array of question and response sets on lifestyle, diet, and nutrition, the UK Biobank cohort presents one of the premier international cohort studies in the 21^st^ century. Moreover, the prospective collection of validated health outcomes, with linkages to national death and cancer registries and hospital episodes statistics for all cohort participants, minimises loss to follow-up and ensures temporality between risk factors and health outcomes.

This study also demonstrates the application and evaluation of ML algorithms following best-practice reporting guidelines for developing prognostic models [24]. Crucially, this includes exploring, understanding and providing detailed information on the characteristics of the cohort and the participants. We have provided the coding structure of each individual variable included in the analysis, documented the steps and sample size when splitting the dataset, and detailed information on how we evaluated performance of each of the algorithms. Uniquely, we have also provided a full hyper-parameters list implemented in grid-search which show exactly how and which parameters fed into the machine-learning models to optimally determine the most accurate models. This extends reporting guidelines to further enhance transparency of ML algorithms.

We purposely used two popular but very different types of ML algorithms, which not surprisingly, have shown variations in the importance of different risk factors. Models using random forests place more importance on continuous or categorical variables with higher number of levels, as they have more opportunities to split [25] within a the decision tree. On the other hand, deep learning using neural network places more emphasis on categorical variables which will produce “latent” classes in the hidden layers that may represent unobserved variables that represent individuals with similar characteristics–an alternative approach to account for unmeasured confounding [26].

Moreover, the speed with which ML models were developed and trained was much faster than traditional epidemiological model building which required a stepwise approach, with clinical interpretation of the relevancy of each variable. Training and validating the ML algorithms took only several hours. In contrast, the final Cox model required over two weeks of researcher time.

The combination of using both techniques may help inform exploration of novel determinants of premature mortality in more causal epidemiological study designs. For instance, we note skin tone was determined to be an important predictor of all-cause mortality in the random forest algorithms. This is interestingly supported by a US study on the spectrum of skin tone and mortality [27]. Both residential air pollution and exposure to hazardous materials, consistent with findings from China [28] and Europe [29], are important variables in the deep learning model. However, they are not statistically significant in the hazard models in either univariate or multivariate analysis.

### Limitations

The primary limitation of ML algorithms, in particular artificial neural networks, is that they are viewed as a “black-box”, which refers to the complexity of how risk factor variables are interacting and what effect they have on the outcome [30]. Whilst many ML algorithms do not produce “effect sizes” such as a hazard ratio from Cox regression, variable importance can still be determined as shown in this study. However, these effect sizes only give an indication of whether there may be a “signal” in the data and not the direction of association, and should thus be interpreted with caution. Further analysis using causal epidemiological study designs is recommended.

ML algorithms, due to large number of risk factors, can also potentially result in over-fitting. This was addressed by active selection of pre-training, hyper-parameter selection, and regularisation of models [31]. For instance, during the grid-search, we sampled variable combinations based on increments of 5 variables for the random forest, and included a hyper-parameter that controls for dropping variables out of the model that do not contribute to minimising the loss function.

The ML algorithms demonstrated in this study were assessing classification accuracy at follow-up, whereas the Cox regression models have an added advantage of assessing time-to-event. Further investigation of ML approaches which can potentially incorporate time-to-event (for instance, deep convoluted neural networks or random survival trees) warrant future exploration and refinement.

Finally, we split the UK Biobank dataset internally for training and testing, an approach commonly used for developing prognostic models [10]. By making predictions on the test cohort which was derived from the overall cohort, we expected the overall accuracy to be higher. The UK Biobank has a “healthy volunteer” bias, with individuals from more educated, less deprived socioeconomic backgrounds, and under-representation of ethnic minorities as shown in the data we presented. Further external validation of the algorithms in this study should be undertaken to increase the generalisability of the findings.

### Future implications

Prognosis research using machine-learning will likely increase exponentially over the next few years, as the techniques used become more commonplace. However, prior to further translation and use in clinical settings, the analytic validity of ML algorithms needs to be established, which requires external validation and replication. Currently, this is difficult to achieve because studies using ML are varied in their nature, purpose and reporting, and are not necessarily led from clinical or health care disciplines. We recognise that a deep learning model with three layers and 50 nodes in each layer, such as the one developed in this study, cannot be completely 100% replicated given the nature of the learning methodology. However, by transparently reporting all the necessary coding structures of the variables, population characteristics, and hyper-parameters for training the models used here, we are confident very similar and comparable models could be developed.

This study has found deep learning and random forest ML algorithms improved accuracy of prediction of premature all-cause mortality in a large middle-aged general population, compared to standard methods. This needs to be further explored in other large databases and in other populations. The intriguing variations in ML model composition may enable new hypothesis generation for potentially significant risk factors that would otherwise not have been detected using standard approaches. Epidemiological studies could then be designed specifically, and powered accordingly, to verify these signals. Future work should also examine other common ML algorithms (e.g. support vector machines, gradient boosting, and probabilistic graphic models), each with their own advantages and disadvantages. For instance, in a two-class prediction problem using data which are free from outliers, structural risk minimisation using support vector machines may be most appropriate.

This study demonstrates the value and exploitation of ML for risk prediction within a traditional epidemiology study design. It illustrates how ML methods might be used in such observational research, and how this might be reported to assist scientific verification and comparison between studies.

## Supporting information

S1 Table Full variable importance rankings scaled for random forest algorithm. (DOCX)

S2 Table Full variable importance rankings scaled for deep learning algorithm. (DOCX)

S3 Table Model discrimination in the validation cohort (n = 125,657) for stratifying outcomes to vascular and non-vascular causes. (DOCX)
