# Supplementary material for: Prediction of premature all-cause mortality: A prospective general population cohort study comparing machine-learning and standard epidemiological approaches
Source: PLoS One. 2019 Mar 27;14(3):e0214365. doi: 10.1371/journal.pone.0214365 (PMC6436798; doi:10.1371/journal.pone.0214365)
Supplement: S1 Table — (DOCX) [file pone.0214365.s001.docx]

| **Overall Rank** | **Variable** | **Mean Decrease in Accuracy** |
| --- | --- | --- |
| 1 | BMI | 45.75 |
| 2 | FEV1 | 42.98 |
| 3 | Waist circumference | 36.61 |
| 4 | Diastolic blood pressure | 36.23 |
| 5 | Systolic blood pressure | 36.23 |
| 6 | Age | 35.36 |
| 7 | Body fat percentage | 30.05 |
| 8 | Cigarettes per day | 23.58 |
| 9 | Prior dx cancer | 21.33 |
| 10 | Gender | 19.92 |
| 11 | Skin tone | 15.90 |
| 12 | Education | 14.77 |
| 13 | Prior dx T2DM | 14.23 |
| 14 | Vegetable consumption | 12.82 |
| 15 | Fruit consumption | 12.54 |
| 16 | Metformin prescribed | 11.11 |
| 17 | Processed meat consumption | 10.70 |
| 18 | Cereal consumption | 9.60 |
| 19 | Fish consumption | 9.60 |
| 20 | Beef consumption | 9.01 |
| 21 | Alcohol consumption | 8.52 |
| 22 | Pork consumption | 8.35 |
| 23 | Aspiring consumption | 8.18 |
| 24 | Ease of skin tanning | 7.89 |
| 25 | Cheese consumption | 7.35 |
| 26 | MET-min per week | 7.31 |
| 27 | Statins prescribed | 6.97 |
| 28 | Digoxin prescribed | 6.92 |
| 29 | Type of milk used | 6.59 |
| 30 | Townsend deprivation score | 6.38 |
| 31 | Vitamin supplements | 5.73 |
| 32 | Prior dx CHD | 4.90 |
| 33 | Sunscreen usage | 4.81 |
| 34 | Warfarin prescribed | 4.46 |
| 35 | Prior dx stroke/TIA | 4.27 |
| 36 | Residential air pollution | 3.92 |
| 37 | Environmental tobacco smoke | 2.91 |
| 38 | Salt added to food | 2.84 |
| 39 | Family history prostate cancer | 2.39 |
| 40 | Oral contraceptives prescribed | 2.28 |
| 41 | HRT prescribed | 1.60 |
| 42 | Prior dx Crohn's disease | 1.52 |
| 43 | Prior dx coeliac disease | 1.14 |
| 44 | Beta-carotene supplements | 0.57 |
| 45 | Smoking | 0.54 |
| 46 | Family history colorectal cancer | 0.53 |
| 47 | Previously had *h. pylori* infection | 0.16 |
| 48 | Prior dx prostate disease | -0.28 |
| 49 | Family history of breast cancer | -0.34 |
| 50 | Blood pressure treatment | -0.57 |
| 51 | Prior dx hyperplasia | -1.26 |
| 52 | Prior dx bowel polyps | -1.47 |
| 53 | Family history of lung cancer | -1.91 |
| 54 | Prior dx thyroid disease | -2.27 |
| 55 | Prior dx COPD | -3.06 |
| 56 | Prior dx acid reflux | -3.61 |
| 57 | Previously had radiotherapy | -4.34 |
| 58 | Job exposure to hazardous materials | -12.32 |
