# Supplementary material for: Prediction of premature all-cause mortality: A prospective general population cohort study comparing machine-learning and standard epidemiological approaches
Source: PLoS One. 2019 Mar 27;14(3):e0214365. doi: 10.1371/journal.pone.0214365 (PMC6436798; doi:10.1371/journal.pone.0214365)
Supplement: S2 Table — (DOCX) [file pone.0214365.s002.docx]

| **Overall Rank** | **Variable** | **Variable importance** |
| --- | --- | --- |
| 1 | Smoking | 0.58 |
| 2 | Age | 0.58 |
| 3 | Prior diagnosis cancer | 0.57 |
| 4 | Alcohol consumption | 0.53 |
| 5 | Digoxin prescribed | 0.52 |
| 6 | Gender | 0.52 |
| 7 | Warfarin prescribed | 0.52 |
| 8 | Townsend deprivation index | 0.51 |
| 9 | Residential air pollution | 0.51 |
| 10 | Prior diagnosis CHD | 0.51 |
| 11 | Statins prescribed | 0.51 |
| 12 | Prior diagnosis COPD | 0.50 |
| 13 | Job exposure to hazardous materials | 0.50 |
| 14 | Education | 0.50 |
| 15 | FEV1 | 0.50 |
| 16 | Sunscreen usage | 0.50 |
| 17 | Blood pressure treatment | 0.50 |
| 18 | Oral contraceptives prescribed | 0.50 |
| 19 | Skin tone | 0.50 |
| 20 | Prior diagnosis bowel polyps | 0.49 |
| 21 | Fish consumption | 0.49 |
| 22 | Vegetable consumption | 0.49 |
| 23 | Beta-carotene supplements | 0.49 |
| 24 | Prior diagnosis thyroid disease | 0.48 |
| 25 | Cigarettes per day | 0.48 |
| 26 | Processed meat consumption | 0.48 |
| 27 | Aspirin Prescribed | 0.48 |
| 28 | Salt added to food | 0.48 |
| 29 | Cereal consumption | 0.47 |
| 30 | Ease of skin tanning | 0.47 |
| 31 | Family history of breast cancer | 0.47 |
| 32 | BMI | 0.47 |
| 33 | Family history of prostate cancer | 0.47 |
| 34 | Cheese consumption | 0.47 |
| 35 | Fruit consumption | 0.47 |
| 36 | Beef consumption | 0.46 |
| 37 | Previously had radiotherapy | 0.46 |
| 38 | Previously had *h. pylori* infection | 0.46 |
| 39 | Prior diagnosis reflux | 0.46 |
| 40 | Waist circumference | 0.46 |
| 41 | Pork consumption | 0.46 |
| 42 | Prior diagnosis Crohn's disease | 0.45 |
| 43 | Body fat percentage | 0.45 |
| 44 | Vitamin consumption | 0.45 |
| 45 | Prior diagnosis prostate disease | 0.45 |
| 46 | Prior diagnosis hyperplasia | 0.45 |
| 47 | Environmental tobacco smoke | 0.45 |
| 48 | HRT prescribed | 0.45 |
| 49 | Prior diagnosis T2DM | 0.44 |
| 50 | Prior diagnosis stroke/TIA | 0.44 |
| 51 | Family history of colorectal cancer | 0.44 |
| 52 | Prior diagnosis Coeliac disease | 0.44 |
| 53 | Type of milk used | 0.44 |
| 54 | Family history of lung cancer | 0.43 |
| 55 | Systolic blood pressure | 0.42 |
| 56 | Metformin prescribed | 0.41 |
| 57 | MET-min per day | 0.40 |
| 58 | Diastolic blood pressure | 0.39 |
