# Supplementary material for: Prediction of premature all-cause mortality: A prospective general population cohort study comparing machine-learning and standard epidemiological approaches
Source: PLoS One. 2019 Mar 27;14(3):e0214365. doi: 10.1371/journal.pone.0214365 (PMC6436798; doi:10.1371/journal.pone.0214365)
Supplement: S3 Table — (DOCX) [file pone.0214365.s003.docx]

| **Validation Cohort Models (n = 125,657)** | **AUC**  **(c-statistic)*** | **Standard Error^$^** | **95% Confidence Interval** |
| --- | --- | --- | --- |
| ***Vascular cause of deaths (Coronary Heart Disease/Cerebrovascular Disease)***  ***N = 1,027*** |  |  |  |
| Age/Gender Cox Model | 0.759 | 0.007 | 0.746 – 0.773 |
| Adjusted Cox Model | 0.837 | 0.006 | 0.825 – 0.848 |
| Random Forest Model | 0.864 | 0.005 | 0.854 – 0.874 |
| Deep Learning Model | 0.864 | 0.005 | 0.854 – 0.873 |
| ***Non-vascular cause of deaths***  ***N = 2,581*** |  |  |  |
| Age/Gender Cox Model | 0.659 | 0.005 | 0.649 – 0.669 |
| Adjusted Cox Model | 0.724 | 0.005 | 0.715 – 0.733 |
| Random Forest Model | 0.746 | 0.005 | 0.737 – 0.754 |
| Deep Learning Model | 0.755 | 0.004 | 0.747 – 0.763 |

*Area under the receiver operating characteristic curve

^$^ Jack-knife procedure to estimate standard errors [20]
